# Geometrical and Electronic Structure of Fluorinated and Non‐Fluorinated Platinum(II) Tetraphenylporphyrin Complexes

**DOI:** 10.1002/cphc.202400973

**Published:** 2025-02-18

**Authors:** Ivan Yu. Kurochkin, Nina I. Giricheva, Valentina A. Ol'shevskaya, Andrey V. Zaitsev, Georgiy V. Girichev, Norbert W. Mitzel

**Affiliations:** ^1^ Department of Physics Ivanovo State University of Chemistry and Technology Sheremetevsky Ave. 7 153000 Ivanovo Russia; ^2^ Institute of Microelectronics Technology and High-purity Materials RAS Chernogolovka, Academician Ossipyan str. 6 Moscow District 142432 Russia; ^3^ Nanomaterial Research Institute Ivanovo State University Ermak Str. 39 153025 Ivanovo Russia; ^4^ A.N. Nesmeyanov Institute of Organoelement Compounds RAS Vaviliva St. 28 119334 Moscow Russia; ^5^ Chair of Inorganic and Structural Chemistry Bielefeld University Universitätsstraße 25 33615 Bielefeld Germany

**Keywords:** Molecular structure, Gas-phase electron diffraction, Macrocyclic ligand, Metal net charge, Platinum

## Abstract

The composition of the saturated vapors of two platinum complexes with the macrocyclic ligands 5,10,15,20‐tetraphenylporphyrin (PtTPP) and 5,10,15,20‐tetrakis(pentafluorophenyl)porphyrin (PtTF_5_PP) and their structures were determined by synchronous gas‐phase electron diffraction/mass spectrometry (GED/MS). These porphyrin complexes are those with the heaviest metal atom in the coordination cavity that have been structurally investigated in the gas phase. The mass spectra confirm the presence of a single molecular form of each, PtTPP (*T*=629 K) and PtTF_5_PP (*T*=597 K). Their structures can serve as references for related complexes in the crystalline state or solutions. Differences between the geometries of PtTPP and PtTF_5_PP in the crystalline and gaseous states include a significant deformation of the tetrapyrrole macrocycle in solid PtTPP. The experimental Pt−N bond lengths of both complexes are in agreement with quantum chemical calculations (DFT/B97D/ECP(Pt)) taking into account relativistic effects. The effect of lanthanide contraction is evident from the similarity of the Pd−N and Pt−N internuclear distances of analogous compounds. The strong electron density transfer from the porphyrin backbone to the metal ion and the resulting low effective positive charge on the platinum atom, studied by NBO and QTAIM methods, helps to rationalize the high catalytic activity of such platinum compounds.

## Graphical abstract

The experimental gas‐phase geometries of two platinum complexes, free from intermolecular interactions, can serve as references for related complexes in the crystalline state or solution. A significant electron density transfer from the porphyrin backbone to the platinum ion make it extremely low positive charged at +0.5 e.

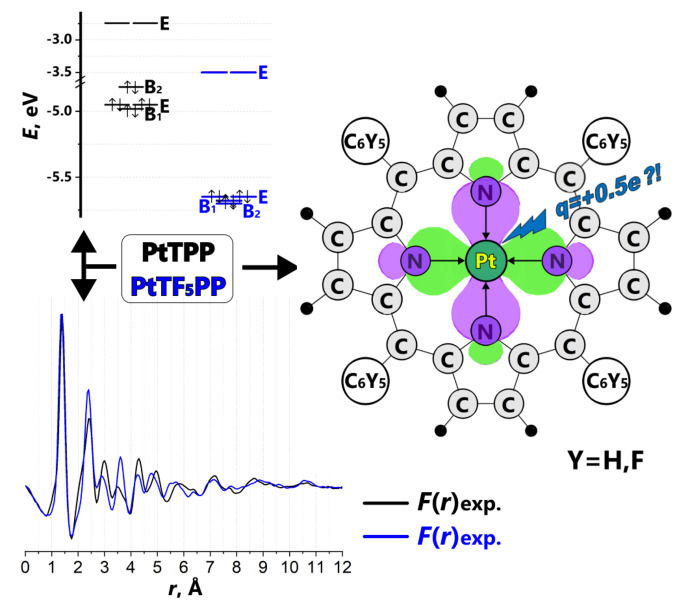

## Introduction

Over the last few decades, metalloporphyrin complexes have been extensively studied both experimentally and theoreticcally.[^1^, ^2^, ^3^] The stability of complexes with different types of metal ions makes it possible to use porphyrin compounds to create highly efficient catalysts, sensors, light‐to‐electricity converters, etc.[^4^, ^5^, ^6^, ^7^, ^8^] Among these applications there is an increasing number of technologies using gas‐phase processes,[^6^, ^7^, ^8^] and it is therefore highly desirable to obtain information on the composition of vapors and the structure of molecular porphyrin complexes in the gas phase.

Few experimental studies by gas‐phase electron diffraction (GED) have been devoted specifically to the geometric structure of free porphyrinoid complexes[^9^, ^10^, ^11^, ^12^, ^13^, ^14^, ^15^] (Sn, Ni, Cu octamethyl porphyrins; Zn, Cu, Co etioporphyrins; Pd, Zn tetraphenylporphyrins) and two molecules with a unsubstituted backbone[^16^, ^17^] (tetrakis(4’‐fluorophenyl) porphyrin and tetraphenyl porphyrin).

Platinum porphyrin complexes are of particular interest for applications in material science because they exhibit intense phosphorescence at room temperature due to the fast intersystem crossing to the triplet state and high emission quantum yields.[^18^, ^19^, ^20^, ^21^] Due to this, these compounds have been widely used for advanced materials, such as organic light‐emitting diodes (OLEDs),[^18^, ^22^] organic solar concentrators,^[23]^ and oxygen sensors.[^24^, ^25^, ^26^] Recently, two photon‐excited phosphorescent dyes based on Pt(II) porphyrins have been designed and applied to direct oxygen imaging in biological systems.[^27^, ^28^, ^29^, ^30^] Platinum tetrabenzoporphyrin and related complexes are also of interest because they produce near‐IR phosphorescence.[^19^, ^31^, ^32^, ^33^, ^34^]

Fluorination of the metalloporphyrins in any position inhibits aggregation processes^[35]^ and increases their catalytic activity^[36]^ and photocatalytic activity in the generation of singlet oxygen (^1^O_2_). The effect of fluorine or chlorine substitution at the phenyl rings of tetraphenylporphyrin (H_2_TPP) has been shown to lead to an increase in the lifetime of the triplet states both in a nitrogen atmosphere and in air and to an increased quantum yield of the singlet oxygen generation in the liquid phase (toluene).[^37^, ^38^] A similar positive effect of fluorine substitution was observed for the porphyrin (H_2_TF_5_PP).^[39]^ Compared with H_2_TPP, a complete fluorination of the phenyl rings in the *meso*‐positions of H_2_TF_5_PP significantly increases the lifetime of the triplet state (by ~1.5) in various media and contributes to an increase in the quantum yield in the of singlet oxygen generation (from 0.6 to 0.8) in dichloromethane.

The aim of this work is to study the effect of fluorine substitution at the phenyl rings on the geometric and electronic structure of Pt(II) tetraphenylporphyrins (PtTPP and PtTF_5_PP). A comparison of the homologous complexes PdTPP^[13]^ and PtTPP should provide information on the effect of lanthanide contraction. The palladium porphyrin complex PdTPP shows an unexpectedly low positive effective charge on the Pd atom,^[13]^ which we expect to be analogous for the PtTPP and PtTF_5_PP complexes. To this end, we study the electronic properties of complexes in terms of natural bond orbital analysis (NBO)^[40]^ and quantum theory of atoms in molecules (QTAIM).^[41]^

## Results and Discussion

### Mass Spectra and Molecular Composition of Saturated Vapors of PtTPP and PtTF_5_PP

To correctly describe the diffraction pattern of a gas electron diffraction (GED) experiment, information about the molecular species present in saturated vapor is necessary. This is provided by a synchronous GED/MS experiment. Table 1 shows the most prominent ions in the mass spectra of PtTPP and PtTF_5_PP recorded during GED experiment. The parent ion has the highest intensity in both cases. The lighter ions are formed by the removal of one or more phenyl groups. In the mass spectra there are also groups of peaks corresponding to doubly charged ions, confirming the tendency for the porphyrins to form the stable parent ions [PtTPP]^2+^ or [PtTF_5_PP]^2+^ at electron ionization.

**Table 1:** Relative abundance *I*_rel._ (%) of molecular and fragment ions in mass spectrum of PtTPP (*U*_ioniz_=50 V, *T*=629 K) and PtTF_5_PP (*U*_ioniz_=50 V, *T*=597 K).

PtTPP			PtTF_5_PP		
Ion	*m/e*	*I*_rel._	Ion	*m/e*	*I*_rel._
[PtTPP]^+^	807	100	[PtTF_5_PP]^+^	1168	100
[PtTPP‐(C_6_H_5_)]^+^	730	41	[PtTF_5_PP–(C_6_F_5_)]^+^	1001	24
[PtTPP‐2(C_6_H_5_)]^+^	653	17			
[PtTPP]^2+^	404	17	[PtTF_5_PP]^2+^	584	17
[PtTPP‐(C_6_H_5_)]^2+^	365	28			
[PtTPP‐2(C_6_H_5_)]^2+^	327	12			

The relative intensities of the ions in the mass spectrum did not change during sample evaporation indicating a stable vapor composition during the effusion experiment. A decrease in ionization voltage is accompanied by a decrease in parent ion fragmentation processes until, at an ionization voltage of 12 V, the parent ion became the exclusive ion seen in the mass spectra. This allows us to conclude, that under the conditions of the GED/MS experiment, the PtTPP and PtTF_5_PP vapors consist of monomeric platinum complexes.

### Conformational Properties of PtTPP and PtTF_5_PP

**Figure 1:**
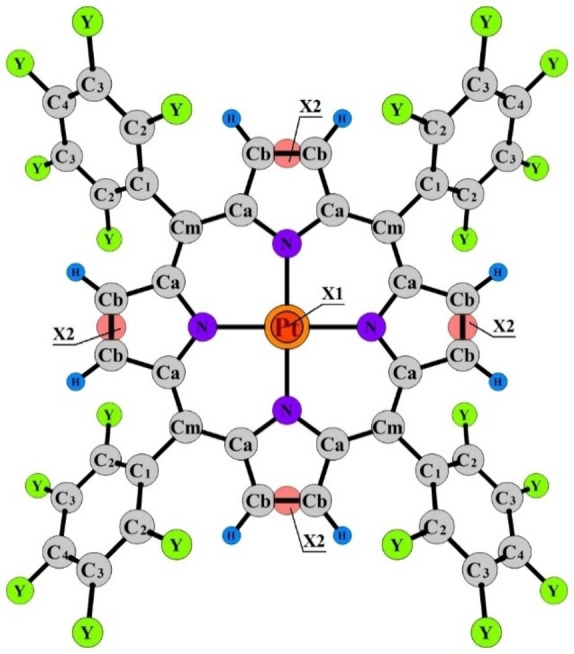
Model of the PtTPP and PtTF_5_PP molecules (Y=H, F) with atom labeling. The position of the phenyl fragments relative to the macrocycle is described by the torsion angle *α*(C_2_−C_1_−C_m_−C_a_); imaginary atom X_1_ (above the plane of the macrocycle by 1 Å), X_2_ – dummy atoms that were used to maintain the molecular symmetry while varying the parameters in the least squares procedure of GED data analysis.

**Figure 2:**
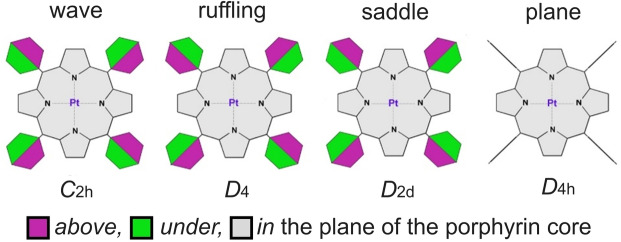
Considered conformations of PtTPP and PtTF_5_PP regarding the orientation of the phenyl groups and macrocycle distortion types.

The presence of substituents in the *meso*‐position that deviate relative to the perpendicular position to the porphyrin backbone leads to the conformational variety described elsewhere.^[13]^ Four structures were considered for PtTPP and PtTF_5_PP (Figure 1). The differences between the configurations and their assignment to the corresponding symmetry groups are shown schematically in Figure 2. The macroheterocycle has a planar structure when the phenyl rings are oriented perpendicular to the cycle (*D*_4h_ symmetry). In other configurations, small non‐planar distortions of the macrocycle occur (wave, ruffling, saddle), which are illustrated in Figure 2.

The relative energies of the conformers, the torsion angles *θ*(C_2_−C_1_−C_m_−C_a_) and *τ*(C_a_−N ⋅ ⋅ ⋅ N−C_a_) characterizing the tilt of the *meso*‐substituents, and the non‐planarity of the macroheterocycle according to DFT calculations, as well as the shape of the macroheterocycle deformation are given in Table 2. Since the out‐of‐plane deformation of the macroheterocycle in the PtTPP and PtTF_5_PP complexes is smaller than in metal‐free H_2_TPP, this indicates an increased rigidity of the macroheterocycle upon formation of Pt−N bonds. It should be noted that in the presented porphyrin complexes the bond length *r*(M−N) slightly exceeds the threshold value of 2.01 Å, which corresponds to the minimum tension of the macrocycle that does not cause its deformation upon the introduction of the metal atom.[^42^, ^43^] This is indicated by the planar molecular structure of platinum(II) porphyrinate in the crystal (Figure S1). That is, in the examples given, the deformation of the macroheterocycle only reflects the influence of the nature and orientation of the *meso*‐substituents. The shape and degree of non‐planar distortion of the porphyrin backbone is determined by the type of symmetry of the conformer, i. e. relative orientation of *meso*‐substituents. The structural models with tilting orientation of the C_6_X_5_ (X=H or F) rings relative to the porphyrin backbone of the PtTPP and PtTF_5_PP molecules demonstrate an energy balance between orbital conjugation of the C_6_H_5_ or C_6_F_5_ rings and the porphyrin backbone on the one hand and steric repulsion of the C_6_X_5_ groups and pyrrole rings on the other hand. The larger size of the C_6_F_5_ groups, compared to C_6_H_5_, increases the steric energy and reduces the deviation of the angle *θ*(C_2_−C_1_−C_m_−C_a_) from 90° in PtTF_5_PP compared with PtTPP.

**Table 2:** Relative energies *▵E*, torsion angles *θ*(C_2_−C_1_−C_m_−C_a_) and *τ*(C_a_−N ⋅ ⋅ ⋅ N−C_a_) for conformers of PtTPP and PtTF_5_PP according to QC calculations.

	PtTPP				PtTF_5_PP			
	*C*_2h_	*D*_4_	*D*_2d_	*D*_4h_	*C*_2h_	*D*_4_	*D*_2d_	*D*_4h_
B3LYP^[a]^	min	min	min	SP4^[b]^	–	–	–	min
*▵E*, kJ/mol	0.1	0.2	0.0	0.2	–	–	–	0.0
*θ*(C_2_−C_1_−C_m_−C_a_), °	82.6	84.4	80.1	90.0	–	–	–	90.0
*τ*(C_a_‐N−N−C_a_), °	0.2	0.1	1.3	0.0	–	–	–	0.0
B97D^[a]^	min	min	min	SP4^[b]^	min	min	min	SP4^[b]^
*▵E*, kJ/mol	1.1	1.7	0.0	6.4	0.2	0.3	0.0	1.9
*θ*(C_2_−C_1_−C_m_−C_a_), °	67.7	68.8	65.3	90.0	74.9	75.3	74.2	90.0
*τ*(C_a_−N−N−C_a_), °	0.7	0.3	4.1	0.0	0.5	0.0	2.0	0.0
Out‐of‐plane MHC distortion shape	wave	ruffling	saddle	planar	wave	ruffling	saddle	planar

[a] for Pt atom: ECP60MDF/aug‐cc‐pVTZ‐PP^44^ were applied. [b] fourth order saddle point.

Predictions of the conformational composition of the compound vapors under consideration using different DFT functionals are not unambiguous in all cases. For PtTPP, two variants of calculations with the B3LYP and B97D functionals lead to the same result at the qualitative level: the presence of three conformers of the molecule and a transition state of *D*_4h_ symmetry. The *D*_2d_ conformer is the minimum in energy. However, for PtTF_5_PP, the use of the B3LYP functional led to a single structure of *D*_4h_ symmetry, while calculations with the B97D functional predicted the presence of three conformers and a highly symmetric transition state (Table 2). The differences in energy and bond lengths of the conformers within one level of QC calculations are insignificant.

Note that the B97D functional takes into account dispersion effects and leads to a greater deviation of the phenyl fragments from the perpendicular position relative to the macroheterocycle (Table 2) and to a greater nonplanar distortion of the macroheterocycle (Table S1). The performed conformational analysis allows us to suggest which molecular forms will be present in the gas phase at elevated temperatures, and what factors need to be taken into account when performing structural analysis of the GED data.

The main geometrical parameters for conformers of PtTPP and PtTF_5_PP, obtained in QC calculations are given in Table S3.

### Molecular Structures of PtTPP and PtTF_5_PP by GED

As follows from Table 2, the difference in conformer energy is significantly lower than the thermal energy of the GED experiment. Moreover, the low value of the relative energy for the *D*_4h_ saddle point indicates the possibility of practically free transitions between stable conformations. Such transitions are also possible in the case where the relative energy of the transition state TS (*D*_4h_) is slightly higher the thermal energy of the GED experiment (in the case of B97D for PtTPP, the predicted Δ*E*_*D*4h_=6.4 kJ/mol vs. *E*_RT_=5.3 kJ/mol). This is because the rotation of two (not four) C_6_H_5_‐rings is sufficient to transition from one stable conformer to another (*D*_2d_ to *C*_2h_/*D*_4_).

The sensitivity of GED data to conformational diversity is shown in Figures S2–S4. They show that the difference between the calculated radial distribution curves *f*(*r*) as well as the theoretical molecular scattering intensities *sM*(*s*) of different conformers is small, since most of the geometry parameters of the conformers are the same, and the non‐planar distortion of the macroheterocycle, as well as the difference in the torsion angle *θ*(C_2_−C_1_−C_m_−C_a_) are small and do not exceed the error in determining the dihedral and torsion angles by the GED method. This allows performing the least squares analysis of GED data using the starting approximation for one conformer. A conformer of *D*_2d_ symmetry was chosen as the geometric model since, formally within the framework of the DFT/B97D method, *D*_2d_ conformers have a minimum energy.

**Figure 3:**
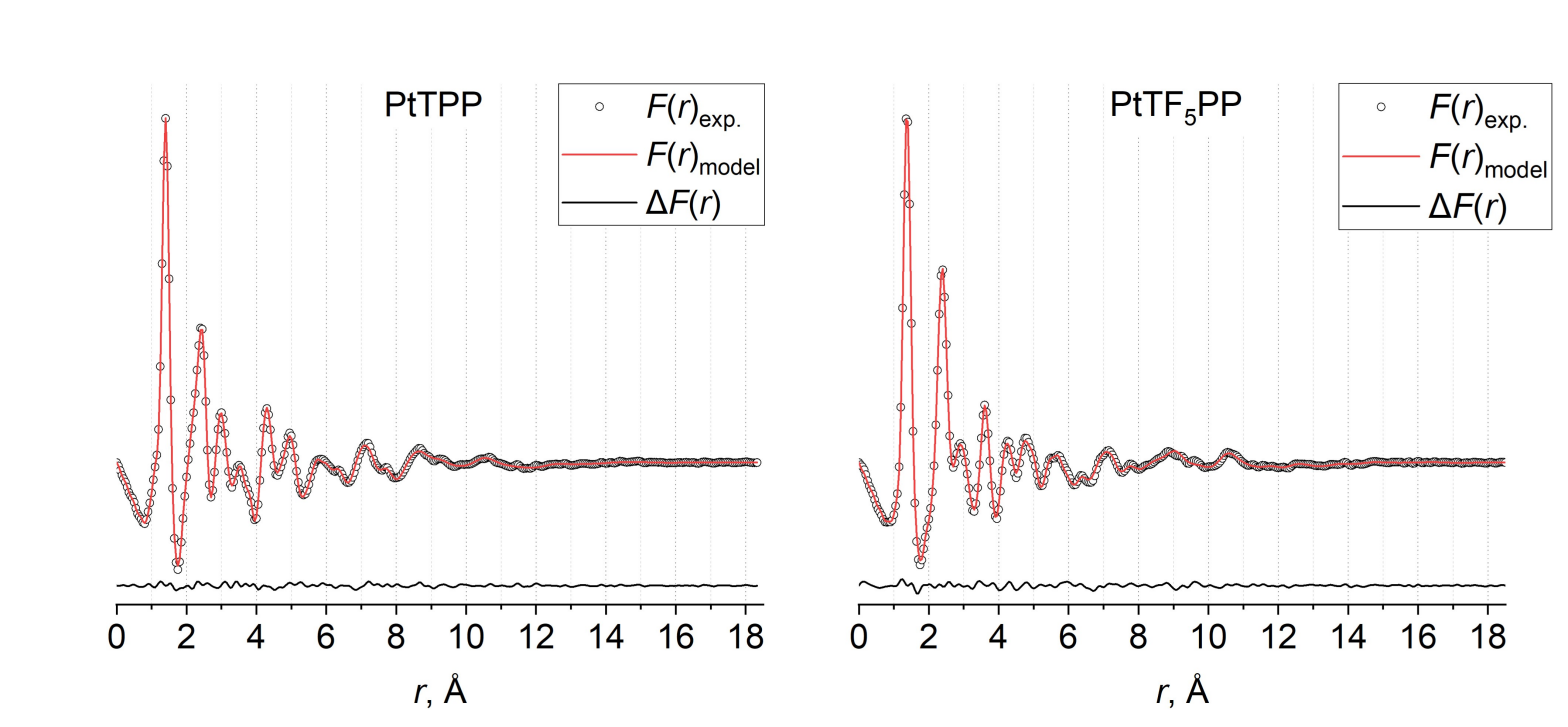
Experimental (circles) and theoretical (solid line) radial distribution functions *f*(*r*) for PtTPP and PtTF_5_PP and residual curves Δ*f*(*r*).

The results of the least square analyses of the GED data for the PtTPP and PtTF_5_PP molecules are presented in Tables 3, S2–S4 and Figure 3. The excellent agreement between the experimental and theoretical radial distribution functions demonstrates the reliability of the obtained main structural parameters of the molecules. For comparison the calculated quantum chemical (QC) and X‐ray diffraction (XRD) parameters (taken from the CSD database^45^) of PtTPP and PtTF_5_PP are given in Table 3.

**Table 3:** Selected geometrical parameters of PtTPP, and PtTF_5_PP obtained by GED,^[a]^ QC and XRD.

Parameters^[b]^	GED^[a]^ *r*_h1_		B3LYP *r*_e_		B97D *r*_e_		XRD (min–max)	XRD (min–max)	XRD (min–max)
	PtTPP	PtTF_5_PP	PtTPP	PtTF_5_PP	PtTPP	PtTF_5_PP	PtTPP^47^	PtTPP^48^	PtTF_5_PP^49^
*r*(Pt−N)	2.025(4)^[b]^	2.032(5)^[b]^	2.027	2.026	2.032	2.030	2.006–2.015(2)	2.009(4)	2.013–2.029(6)
*r(*C_a_−N)	1.383(3)	1.378(3)	1.375	1.373	1.383	1.380	1.380–1.391(4)	1.373–1.380(7)	1.351–1.382(10)
*r*(C_a_−C_b_)	1.440(3)	1.437(3)	1.439	1.439	1.440	1.440	1.429–1.445(4)	1.436–1.440(7)	1.418–1.437(12)
*r*(C_b_−C_b_)	1.359(3)	1.354(3)	1.352	1.351	1.360	1.359	1.344–1.350(5)	1.348(8)	1.331–1.342(12)
*r*(C_m_−C_a_)	1.398(3)	1.393(3)	1.393	1.391	1.399	1.395	1.390–1.407(5)	1.388–1.402(7)	1.367–1.397(12)
*r*(C_m_−C_Ph1_)	1.491(3)	1.489(3)	1.497	1.495	1.492	1.492	1.496–1.505(4)	1.489(7)	1.470–1.490(12)
*r*(C_1_−C_2_)	1.403(3)	1.397(3)	1.396	1.392	1.403	1.399	1.379–1.400(6)	1.383–1.395(7)	1.377–1.409(14)
*r*(C_2_−C_3_)	1.396(3)	1.395(3)	1.390	1.388	1.397	1.397	1.384–1.395(5)	1.395–1.399(7)	1.364–1.399(15)
*r*(H−C_b_)	1.090(5)	1.081(3)	1.075	1.076	1.080	1.081	0.840–1.050(40)	0.950	0.949–0.951
*r*(C−C)_ave_	1.399(3)	1.396(3)	1.392	1.389	1.399	1.398	1.384(6)	1.391(7)	1.371(15)
*r*(C−F)_ave_	–	–	–	1.334	–	1.340	–	–	1.342(12)
*α*(Pt−N−C_a_)	126.7(2)	126.6(3)	126.6	126.6	126.7	126.8	126.3–127.9(2)	126.2–126.3(3)	125.6–127.2(8)
*α*(C_a_−N−C_a_)	106.4(3)	106.7(5)	106.8	106.7	106.6	106.4	105.4–106.3(3)	107.3(5)	106.0–108.4(8)
*α*(N−C_a_−C_b_)	109.5(3)	109.3(5)	109.3	109.4	109.4	109.6	108.9–110.3(3)	108.7(4)	107.7–109.4(8)
*α*(C_a_−C_m_−C_a_)	124.5(1)	124.7(1)	124.3	124.9	124.5	125.2	123.2–124.6(3)	123.4(5)	124.2–126.8(9)
*α*(C_1_−C_2_−C_3_)	120.5(3)	121.6(1)	120.7	122.0	120.6	121.9	119.1–121.0(4)	120.0–121.0	120.9–121.9(10)
*θ*(C_2_−C_1_−C_m_−C_a_)	71(4)	79(^−5^_+27_)	80.1	90.0	65.3	74.2	53.5–84.3(5)	78.2–78.5(7)	75.0–87.7(10)
*θ*(C_m_ ⋅ ⋅ ⋅ C_m_ ⋅ ⋅ ⋅ C…C_m_)	0	0	0	0	0	0	5.2(1)	18.5	0.0(2)
*τ*(C_b_−C_b_…C_b_−C_b_)	0	0	0	0	0	0	6.5–7.6(3)	26.7	0.0(9)
*ϕ*(N−C_a_ ⋅ ⋅ ⋅ C_a_−N)	4.7	2.3	1.5	0	4.7	2.3	4.0–7.4	3.0	0.6(1)
*r*(C_m_…C_m_)	6.91(1)	6.89(2)	6.89	6.87	6.91	6.89	6.858/6.916	6.805	6.834/6.857
*R*_f_, %	3.9	4.4					2.1	2.1	4.9

[a] results of GED refinement based on starting structure from B97D calculations (*D*_2d_); results of refinement based on B3LYP calculations are shown in Table S1. [b] Uncertainties given in parentheses were taken for distances as: [(2.5*σ*_LS_)^2^+*σ* ^2^_scale_]^1/2^, *σ*_scale_=0.002*r*, *σ*_LS_ is a standard deviation in the least‐squares refinement; 3*σ*_LS_ for bond and torsion angles.

The experimental macroheterocycle backbone parameters for both PtTPP and PtTF_5_PP are in agreement with each other and with the calculated parameters within experimental uncertainty. The exception is the torsion angle C_2_−C_1_−C_m_−C_a_. As predicted by QC calculations, the experimental value of *θ*(C_2_−C_1_−C_m_−C_a_) for PtTF_5_PP is closer to 90° than for PtTPP.

**Figure 4:**
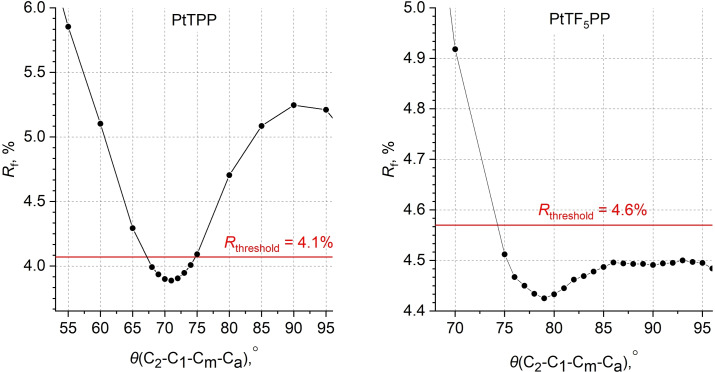
The ratio of disagreement factors *R*_f_ as a function of torsion angle *θ*(C2−C1−C_m_−C_a_), responsible for the rotation of C_6_H_5_‐ and C_6_F_5_‐groups in PtTPP, and PtTF_5_PP. *R*_threshold_ – uncertainty according to Hamilton's statistical criterion^[46]^ at 0.05 significance level.

To determine the sensitivity of GED data to the rotation of the *meso*‐substituents relative to the macroheterocycle, the dependence of the disagreement factor *R*_f_ on the angles *θ*(C_2_−C_1_−C_m_−C_a_) was determined (Figure 4) under relaxation of the other geometric parameters. In the case of PtTPP, the rotation of the phenyl rings to perpendicular position leads to a significant increase in *R*_f_, which exceeds the threshold value, calculated using Hamilton's criterion for a significance level of 0.05. Thus, using this criterion, the torsion angle *θ*(C_2_−C_1_−C_m_−C_a_) for the PtTPP complex can be estimated to be 71(4)°. In the case of PtTF_5_PP, a different picture is observed: rotation of the phenyl rings (*θ*) in the range between 74 and 106° weakly affects the *R*_f_ value, that is they remain below the threshold value.

This experimental fact corresponds to the calculated (B97D) ratio of the energy of the conformers and the transition state (Table 3), which shows that at the temperature of the GED experiment (*E*_RT_=5.3 kJ/mol) the over‐barrier transitions between the conformers are possible. This leads to an effective average value of the torsion angle of 79(5)° (Table 3).

### Structure Differences Between Gas and Crystal Phases

When using a solid‐state structure determined by XRD to search for stereochemical regularities, one must keep in mind that such a structure may be far from that of an isolated molecule due to collective interactions in the crystal, as well as due to the peculiarities of the scattering of X‐rays in the diffraction experiment. Considering the changes in the structure of compounds during the crystal‐gas transition is important for structural chemistry, since it allows not only to evaluate the degree of deformation of the molecule arising due to intermolecular interaction forces, and to determine the least‐rigid fragments, but also to calculate the energy of structural deformation (*E*_def_=*E*_cryst_–*E*_gas_). The latter, together with the energy of the crystal lattice, makes it possible to estimate the sublimation enthalpy Δ*H*_sub_, or changes in the value of Δ*H*_sub_ if the compound has several polymorphs.

For platinum complexes, the Cambridge Structural Database (CSD)^[45]^ contains information on two types of PtTPP structures[^47^, ^48^] and one PtTF_5_PP.^[49]^ For PtTPP the structure of the complexes of the triclinic (*T*=150 K)^[47]^ and tetragonal (*T*=100 K)^[48]^ crystal phases differ significantly (Table 3, Figures S6–S9). In the first crystal,^[47]^ the direction of rotation of four *meso*‐substituents corresponds to a conformer with *D*_2d_ symmetry. However, all angles *θ*(C_a_−C_m_−C_1_−C_2_) have different values from 54° to 82°, resulting in an asymmetric non‐planar saddle distortion of the macroheterocycle with torsion angles *ϕ*(N−C_a_ ⋅ ⋅ ⋅ C_a_−N) from 4° to 8°, dihedral angles between the planes of opposite pyrrole fragments *τ*(C_b_−C_b_ ⋅ ⋅ ⋅ C_b_−C_b_)=6–8°. However, the PtN_4_ fragment retains an almost flat structure.

In a crystal of tetragonal syngony,^[48]^ the complex is more symmetrical with angles *θ*(C_a_−C_m_−C_1_−C_2_) = 80° (the direction of rotation of the *meso*‐substituents corresponds to *D*_4_ symmetry), with a flat PtN_4_ structure fragment and large dihedral angles *τ*(C_b_−C_b_ ⋅ ⋅ ⋅ C_b_−C_b_)=26.7° (ruffling distortion of the macroheterocycle, Figures S8 and S9). Overall, there is a significant but different distortion of the structure of the PtTPP complex in the two crystals compared to the structure of the free molecule determined by GED. In the PtTPP crystal,^[47]^ molecules enter into three types of interactions: a) T‐shaped stacking between two C_6_H_5_ rings (2.82, 3.02, 3.50 Å), b) aryl‐stacking between pyrrole rings (3.97 Å), and c) C−H ⋅ ⋅ ⋅ N interactions between the C_6_H_5_ rings and the nitrogen atoms (2.64, 2.68, 2.74 Å) of the macroheterocycle. The four C_6_H_5_ substituents of the molecule in the PtTPP crystal^[47]^ participate in the listed types of interactions in a different combination, which leads to a large difference in the angles *θ*(C_a_−C_m_−C_1_−C_2_) and a significant distortion of the macrocycle. In the crystal,^[48]^ there are only two types of interactions between molecules: a) dominant C−H ⋅ ⋅ ⋅ N interactions (2.66, 3.18 Å), and b) aryl‐stacking between pyrrole rings (4.02 Å).

In the case of PtTF_5_PP,^[49]^ the crystal is characterized by tetragonal syngony at 130 K. The PtTF_5_PP complex has an almost flat macroheterocycle structure (*ϕ*(N‐C_a_ ⋅ ⋅ ⋅ C_a_−N)=0.6°). As shown in Figure S10, the PtTF_5_PP molecules in the crystal^[49]^ enter into a large number of close intermolecular F ⋅ ⋅ ⋅ F interactions (2.8–2.9 Å). Until recently, perfluoroarene ⋅ ⋅ ⋅ perfluoroarene interactions were considered as unusual,^[50]^ but meanwhile there are many examples confirming the presence of attractive forces between such units.[^51^, ^52^, ^53^, ^54^] In the present cases, quantum chemical calculations (DFT/PBE)^[55]^ predicted that the electrostatic repulsion F ⋅ ⋅ ⋅ F is not so large and its contribution to the total interaction energies of molecular pairs in crystals was less than 15 %, and the major contribution was from the attraction forces between fluorine atoms, determined by the van der Waals component. Moreover, within the framework of QTAIM, bond critical points were found between the nuclei of F atoms (the electron density in these points varies from 0.003 to 0.004 au),^[56]^ emphasizing the role of weak F ⋅ ⋅ ⋅ F contacts in the supramolecular architecture of the crystals. In the PtTF_5_PP crystal^[49]^ the relative orientation of C_6_F_5_ groups corresponds to the *C*_2h_ symmetry of the complex with torsion angles *θ*(C_a_−C_m_−C_1_−C_2_) of 75° and 88° (weak wave‐like distortion of macroheterocycle, Figures S10–13). The structures of the PtTF_5_PP complex (in contrast to PtTPP) in the crystal and gas phases are very similar, despite the large number of close contacts, which indicates a greater rigidity of the perfluorinated complex compared to the non‐fluorinated one. In both PtTF_5_PP and PtTPP, the central PtN_4_ fragment is flat, despite different deformations of the periphery.

Thus, macroheterocycle distortions in solid PtTPP (ruffling, saddle) and PtTF_5_PP (wave) correspond to their distortions in certain conformers of the free molecules (Figure 2). Experimental data for the free molecules make it possible to estimate the direction of distortions in complexes resulting from intermolecular interactions in the crystal.

### Influence of the Nature of the Metal Atom and *Meso*‐Substituents on the Distribution of Electron Density in the Porphyrin Backbone

In order to assess the effect of various types of substitution on the electronic structure of the Zn, Pd, and Pt porphyrin complexes, we performed NBO and QTAIM analyses. The results are given in Table 4.

**Table 4:** Results of NBO and QTAIM analysis (B97D) for various metal complexes MTPP (M: Zn, Pd, Pt) and PtP, PtTF_5_PP. Listed are bond lengths *r*, net charges *q*, bond orders *P*, electron density Laplacians ∇^2^ *ρ*(**r**) at the bond critical points of the M−N and N−C_a_ bonds, and delocalization indices *DI*.

Compound	ZnTPP	PdTPP	PtTPP	PtP	PtTF_5_PP
*E*_HOMO_, eV	−4.6	−4.8	−4.8	−5.1	−5.6
*E*_LUMO,_ eV	−2.8	−2.8	−2.8	−2.8	−3.5
▵*E*_HOMO–LUMO_, eV	1.8	2.0	2.1	2.3	2.2
*r*(M−N), Å	2.057	2.036	2.032	2.032	2.030
*r*(N−C_a_), Å	1.377	1.379	1.383	1.380	1.380

NBO					
*q*(M)	1.196	0.529	0.515	0.520	0.531
*q*(N)	−0.598	−0.426	−0.425	−0.429	−0.420
electron configuration of M	4 s^0.44^3d^9.97^4p^0.39^	5 s^0.38^ 4d^8.84^ 5p^0.24^	6 s^0.56^ 5d^8.66^6p^0.24^	6 s^0.56^ 5d^8.66^6p^0.24^	6 s^0.56^5d^8.66^ 6p^0.24^
*P*(M−N)	0.32	0.49	0.55	0.55	0.55

QTAIM					
*q*(M)	1.211	0.769	0.780	0.785	0.798
*q*(N)	−1.121	−1.041	−1.034	−1.042	−1.033
∇^2^ *ρ*(**r**) (M−N)	0.27	0.35	0.36	0.36	0.36
*DI*(M/N)	0.45	0.76	0.85	0.85	0.85
∇^2^ *ρ*(**r**) (N−C_a_)	−0.94	−0.90	−0.93	−0.90	−0.90

The series ZnTPP–PdTPP–PtTPP allows us to monitor the influence of the nature of the central atom on the parameters of the M−N bond. The electronic configuration of the M atom in the complexes (Table 4) shows that the 3 d shell of Zn is completely filled, and in the Pd and Pt complexes the central atoms exhibit electron‐withdrawing properties, also due to the *n*d atomic orbitals (AO) in contrast to the Zn complex.

The charge on the central atom is very different from +2. Between the M and N atoms there are strong donor‐acceptor interactions involving the lone pairs of electrons (LP) at the nitrogen atoms, LP(N), and the free AOs of the central atom, which stabilizes the complex. The transfer of electron density from the LP(N) is much greater in the Pd and Pt complexes than in the Zn complex, so the positive charge on these atoms is significantly less than on Zn. This is also expressed by the bond order *P*(M−N) and the delocalization index *DI*(M/N), which show that the Zn−N bond is weaker than Pd−N and Pt−N bonds.

A positive value of the Laplacian ∇^2^ *ρ*(**r**) (M−N) indicates the predominantly ionic nature of the M−N bonds. A change in *P*(M−N) indicates an increase in the covalent component, and a change in the difference in charges on the M and N atoms *q*(M)−*q*(N) indicates a decrease in the ionic component of the M−N bond along the series ZnTPP–PdTPP–PtTPP.

The similarity of the geometric and electronic parameters of the Pd−N and Pt−N bonds indicates the manifestation of the lanthanide contraction effect observed during the transition from compounds of 4d^n^ to 5d^n^ elements. The 4f^14^ electron shell of the Pt atom weakly shields the nuclear charge, leading to an increase in the effective nuclear charge and a decrease in the electron shell effective size. The effective radius of the Pt atom is very close to that of the Pd atom: 1.40 versus 1.43 Å, respectively.

The influence of the nature of the *meso*‐substituent can be traced along the series PtTPP–PtP–PtTF_5_PP (Table 4). It can be seen that the properties of the Pt−N bond do not change. There are only minor changes in the geometry of the macroheterocycle (Table 3). The most pronounced difference is in the relative orientation of the planes of the substituents and the macroheterocycle. Due to their larger size and higher energy of steric interaction with pyrol fragments, the C_6_F_5_ substituents occupy a position almost perpendicular to the macroheterocycle plane, and for the C_6_H_5_ substituents the torsion angles *θ*(C_a_−C_m_−C_1_−C_2_) differ markedly from 90° (Table 3).

**Figure 5:**
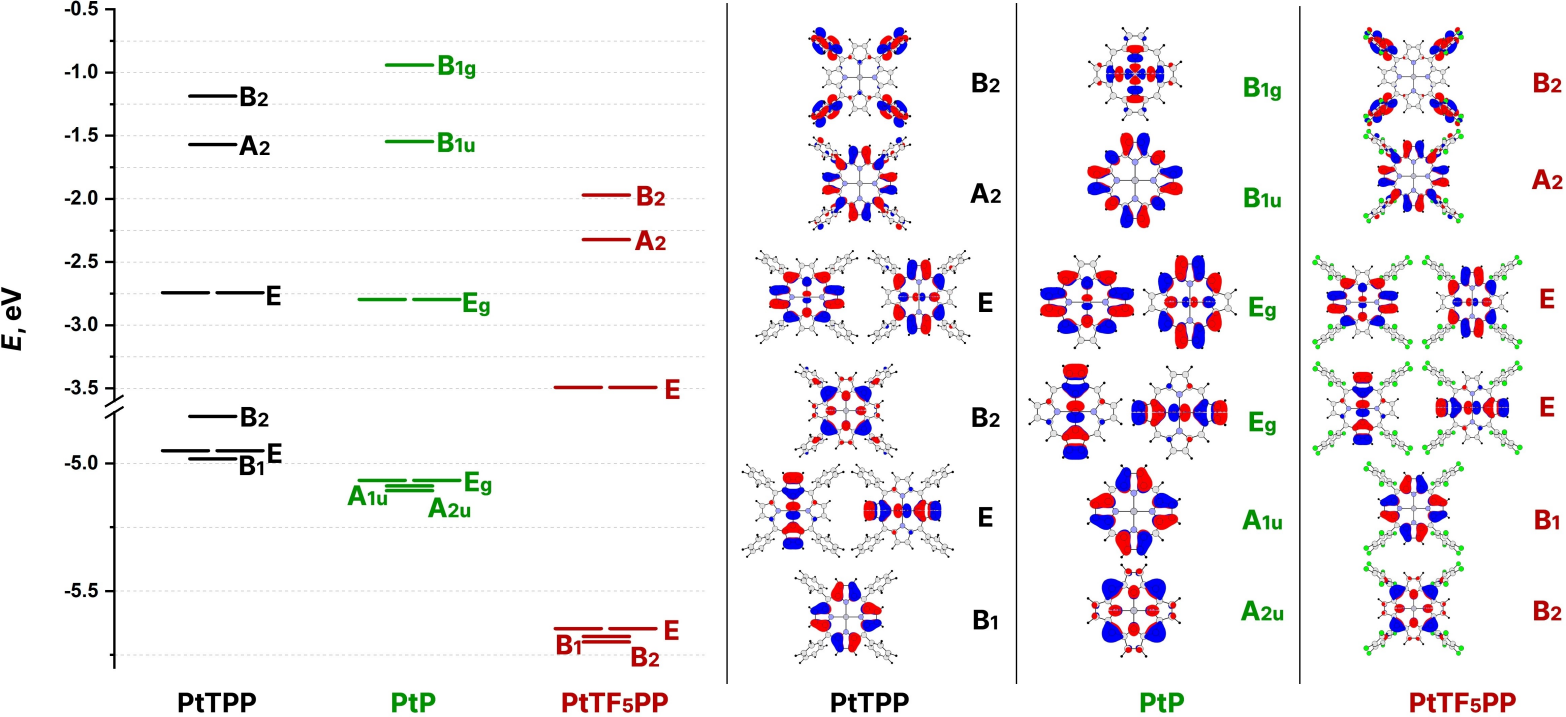
Frontier orbitals for PtP, PtTPP, PtTF_5_PP.

The greatest effect of introducing *meso*‐substituents into the PtP complex is the difference in the energy of the frontier orbitals and the symmetry of the HOMO. Six MOs for three complexes are shown in Figure 5. A comparison of the *E*_HOMO_ and *E*_LUMO_ of the PtP with those of the PtTPP and PtTF_5_PP complexes demonstrates the different nature of the C_6_H_5_ and C_6_F_5_ substituents. An increase in the HOMO energy in PtTPP indicates an electron‐donating effect of C_6_H_5_ substituents, while a decrease in LUMO energy in PtTF_5_PP indicates an electron‐withdrawing effect in the case of C_6_F_5_ substitution.

The LUMO in the three complexes are doubly degenerate, to which 4d_xz_ and 4d_yz_ AOs at platinum contribute, indicating the possibility of an interaction of the central atom of the complex with electron donor substances. Since the LUMO energy of PtTF_5_PP is lower, the probability of such an interaction may be higher than that of PtTPP.

The HOMOs of the PtP and PtTF_5_PP complexes are doubly degenerate and represent a linear combination of p_π_ AO of the pyrrole rings and the d_xz_/d_yz_ AOs of platinum. The HOMO of the PtTPP complex has B_2_ symmetry, and their electron density is concentrated on the C_m_ and N atoms, reflecting the electron‐donating nature of the phenyl substituents.

As can be seen from Figure 5, the AOs of the *meso*‐substituents are not included in the composition of the near‐frontier MOs, however, the different symmetry and energy of the HOMO along the series PtTPP–PtP–PtTF_5_PP shows the possibility to modify the electronic properties of these complexes with the introduction of substituents of different nature, despite the small change in their geometric structure.

## Conclusions

The saturated vapors of PtTPP (*T*=637 K) and PtTF_5_PP (*T*=597 K) were studied by GED/MS and the presence of a monomeric molecular form in their vapors was established. Conformational analyses performed by the DFT method (B3LYP and B97D) showed that the PtTPP and PtTF_5_PP molecules can have conformers that differ in the relative orientation of the four *meso*‐substituents. The conformers have similar energies and low transition barriers between them, which can be overcome at the sublimation temperature of these compounds. The basic geometric parameters of macroheterocycle backbone in the conformers are virtually the same.

The structure of the free PtTPP and PtTF_5_PP molecules was determined using the GED method. The experimental values of bond lengths and bond angles are close to the calculated ones and practically do not depend on the nature of the C_6_H_5_ and C_6_F_5_ *meso*‐substituents. The difference lies mainly in the value of the torsion angle between the planes of the *meso*‐substituents and the macroheterocycle backbone. The larger size of the C_6_F_5_ substituents in PtTF_5_PP corresponds to a greater steric repulsion of the substituent from the macroheterocycle backbone and a close to orthogonal relative orientation of their planes.

An analysis of the differences between the geometry of the PtTPP and PtTF_5_PP complexes in the crystalline and gaseous states shows a significant deformation of the macroheterocycle in the PtTPP crystals.

The application of the NBO and QTAIM methods to the series ZnTPP–PdTPP–PtTPP showed that the Zn−N coordination bond is significantly weaker than the Pd−N and Pt−N coordination bonds; there is an increase in the covalent and a decrease in the ionic component of the M−N bond along the series ZnTPP–PdTPP–PtTPP.

The effect of lanthanide contraction in Pt complexes found experimental confirmation, by the similarity of the Pd−N and Pt−N internuclear distances. To assess the influence of the nature of the *meso*‐substituent, the series PtTPP–PtP–PtTF_5_PP was considered. The different symmetry and energy of the HOMO in this series indicate the possibility of modifying the electronic properties of the complexes with the introduction of substituents of different nature, despite a small change in the geometric structure of the macroheterocycle backbone.

The obtained geometric and electronic characteristics of PtTPP and PtTF_5_PP complexes free from collective interactions are reference values for establishing “structure‐property” correlations.

## Experimental

### Sample

The synthesis of the samples of PtTPP and PtTF_5_PP was performed according a reported protocol.^[57]^

### Synchronous Gas‐Phase Electron Diffraction/Mass Spectrometry Experiment (GED/MS)

The diffraction patterns and mass spectra of PtTPP and PtTF_5_PP, respectively, were recorded simultaneously on an EMR‐100/APDM equipment.[^58^, ^59^, ^60^] The samples were evaporated from a cylindrical molybdenum effusion cell. The size of the effusion channel was 0.5×1.6 mm (diameter×length). The ratio of evaporation surface to effusion orifice square was about 400. Detailed experimental conditions of the GED/MS experiment are given in Table S5.

Accurate wavelengths of electrons were calibrated using polycrystalline zinc oxide. The optical densities of the diffraction patterns were measured by a modified MD‐100 (Carl Zeiss, Jena) microdensitometer^[61]^ with a step size of 0.1 mm along diagonal. A 10×130 mm region was scanned; the number of equidistant scan lines was 33.

### Computational Details

The DFT theory level often turns out to be the most optimal in the ratio of computational cost and quality of calculated parameter values as practice shows for such large structures. DFT functionals B3LYP[^62^, ^63^] and B97D[^64^, ^65^] (without and taking into account dispersion corrections) in combination with basis sets cc‐pVTZ^[66]^ for atoms C, N, H, F, for Pt atom: ECP60MDF/aug‐cc‐pVTZ‐PP^[44]^ taking into account the relativistic effect were applied. PtTPP and PtTF_5_PP molecules can have several conformers (see Figure 2 and Table 2) For each conformer, geometry optimization and calculations of the force field in a harmonic approximation were performed. Features of the electronic structure were studied by the QTAIM and NBO methods.

### Structural Analysis GED

Structural analyses were performed by the UNEX program package.^[67]^

Processing the electron diffraction data involves solving the inverse problem. This suppose an creation of geometrical models, the parameters of which will be refined to achieve minimal disagreement between theoretical and experimental molecular scattering intensity *sM*(*s*) using the least‐squares method (LS) according to the equation:

$$Q_{M}=\sum_{i}^{n}\omega_{i}{\left[sM_{\exp}\left(s_{i}\right)-k_{M}sM_{\mathrm{theor}}\left(s_{i}\right)\right]}^{2}=min$$

Models of the PtTPP and PtTF_5_PP molecules with atom labeling are shown in Figure 1. When refining the GED data, several variants of starting geometries and force fields of PtTPP and PtTF_5_PP, obtained from both the B97D and B3LYP calculations, were tested. All conformers (Figure 2) were considered.

The need to test several initial models is due to the fact that the theoretical approximations used (B97D, B3LYP) can estimate the orientation of *meso*‐substituents and the degrees of macrocycle distortion differently.^[17]^

Vibrational amplitudes and corrections ▵*r*=*r*_h1_–*r*_α_ were calculated based on harmonic force fields calculated for both functionals B97D and B3LYP at the experimental temperatures by VibModule^[68]^ program.

Eight geometric parameters for each PtTPP and PtTF_5_PP molecule were varied independently during the least squares analysis of the GED data, along with 16 groups of vibration amplitudes. A detailed parameter list is provided in the Supporting Information (Table S6).

## Author Contributions

Conceptualization: G.V.G., N.I.G., N.W.M.; methodology: G.V.G., N.I.G., I.Yu.K.; investigation: I.Yu.K., N.I.G., V.A.O., A.V.Z, and G.V.G.; data analysis: I.Yu.K., N.I.G., V.A.O.; resources: N.I.G., G.V.G., N.W.M.; writing, original draft preparation: I.Yu.K., N.I.G., G.V.G., writing, review and editing: G.V.G., N.W.M.; visualization: I.Yu.K., N.I.G.; supervision: G.V.G., N.W.M.; project administration: G.V.G., N.W.M.; funding acquisition: G.V.G., N.W.M.

## Conflict of Interests

The authors declare no conflict of interest.

1

## Supporting information

As a service to our authors and readers, this journal provides supporting information supplied by the authors. Such materials are peer reviewed and may be re‐organized for online delivery, but are not copy‐edited or typeset. Technical support issues arising from supporting information (other than missing files) should be addressed to the authors.

Supporting Information

## Data Availability

The data that support the findings of this study are available in the supplementary material of this article.
