# Supplementary material for: Geometrical and Electronic Structure of Fluorinated and Non‐Fluorinated Platinum(II) Tetraphenylporphyrin Complexes
Source: Chemphyschem. 2025 Feb 18;26(9):e202400973. doi: 10.1002/cphc.202400973 (PMC12058241; doi:10.1002/cphc.202400973)
Supplement: Supplementary file 1 — Supporting Information [file CPHC-26-e202400973-s001.pdf]

ChemPhysChem

Supporting Information

Geometrical and Electronic Structure of Fluorinated and Non-Fluorinated Platinum(II) Tetraphenylporphyrin Complexes

Ivan Yu. Kurochkin, Nina I. Giricheva, Valentina A. Ol'shevskaya, Andrey V. Zaitsev, Georgiy V. Girichev, and Norbert W. Mitzel*

Supplementary information

Geometrical and Electronic Structure of Fluorinated and Non-fluorinated Platinum(II) Tetraphenylporphyrin Complexes

Ivan Yu. Kurochkin[a], Nina I. Giricheva[b], Valentina A. Ol'shevskaya[c], Andrey V. Zaitsev[c], G. V. Girichev[a], Norbert W. Mitzel[d*]

Table S1. Conditions of simultaneous experiments on EMR-100/APDM-1

	PtTPP		$PtTF_5PP$	
Nozzle-to-plate distance, mm	338	598	338	598
Number of recorded films	6	6	6	6
Primary electrons beam current, μA	1.49	0.96	2.13	0.98
Wavelength of electrons, Å	0.04003(4)	0.03976(3)	0.042354(4)	0.040727(4)
Temperature of effusion cell, K	636(5)	638(5)	593(5)	600(5)
Exposure time, s	150	100	70	75
Residual gas pressure, Torr:				
-in the diffraction chamber	$1.6 \cdot 10^{-6}$	$1.8 \cdot 10^{-6}$	$1.2 \cdot 10^{-6}$	$1.8 \cdot 10^{-6}$
-in mass spectrometric block	$6.0 \cdot 10^{-7}$	$5.8 \cdot 10^{-7}$	$4.0 \cdot 10^{-7}$	$4.0 \cdot 10^{-7}$
Scattering angles range / Δs, Å^{-1}	4.2-29.3/0.1	1.3-16.4/0.1	4.0-28.0/0.1	1.6-16.4/0.1
Ionization voltage, V	50	50	50	50

Table S2. List of Z-matrix parameters for PtTPP and $PtTF_5PP$ molecules. Atom numbering is shown in Figure 2.

$PtTY_5PP$ (Y = H, F)
**14 type of distances:**<br>Gr. 1: *r*(Pt-N)<br>Gr. 2: *r*(C_a-N), *r*(C_m-C_a), *r*(X_2-C_b), *r*(X_2-C_a), *r*(C_1-C_2), *r*(C_2-C_3), *r*(C_3-C_4), *r*(C_1-C_m), *r*(C_2-Y), *r*(C_3-Y), *r*(C_4-Y), where Y=F in $PtTY_5PP$<br>Gr. 3: *r*(C_2-Y), *r*(C_3-Y), *r*(C_4-Y), *r*(C_b-H), where Y=H in PtTPP<br>**12 bond angles**<br>Gr. 4: α(C_a-N-Pt)<br>Gr. 5: α(C_2-C_1-$C_{2'}$), α(C_3-C_2-C_1)<br>Gr. 6: α(C_a-C_b-H)<br>Gr. 7: α(C_1-C_2-Y), α(C_2-C_3-Y), α(C_3-C_4-Y)<br>Fixed: α(Pt-N-C_a) (89.8^0/90.2^0), α(C_2-C_1-C_m) (~121^0), α(C_1-C_m-Pt) (180^0), α(C_b-X_2-Pt) (90^0)<br>**16 torsion angles**<br>Gr. 8: θ(C_2-C_1-C_m-C_a)<br>Fixed: θ(N-Pt-X_1-N) (90^0), θ(C_1-C_m-Pt-C_m) (180^0), θ(C_b-X_2-Pt-C_a) (~3^0), θ(C_b-X_2-Pt-C_b) (180^0), , θ(C_a-N-Pt-X_1) (87.7^0/93.3^0), θ(X_2-C_a-C_a-Pt) (~176^0), θ(C_m-C_a-C_a-N) (~178^0), θ(C_2-C_1-C_2-C_m) (180^0), θ(C_3-C_2-C_1-C_2) (~0^0), θ(C_4-C_3-C_3-C_2) (~180^0), θ(Y-C_2-C_1-C_3) (~180^0), θ(Y-C_3-C_2-C_4) (~180^0), θ(Y-C_4-C_3-C_3) (180^0), θ(H-C_b-C_a-C_b) (~179^0)

The parameters varied during the GED structural analysis are listed in the "Gr." groups. For parameters that are in the same group, the QC differences between them are retained. Parameters that are included in the Z-matrix but were not varied in the final version of the structural analysis are labeled as "Fixed" (Fixed at QC values). Differences between nonequivalent structural parameters of the same type were fixed at the calculated values. The structure of D_{2d} symmetry was chosen as start for both molecules.

Table S3. The main geometrical parameters of $PtTY_5PP$ (Y = H, F) obtained in QC calculations that characterize the dimensions and non-planarity of the porphyrin backbone. The atom numbering is shown in Figure 1 (main text).

B97D				B3LYP			
**PtTPP**	C_{2h}	D_4	D_{2d}	**PtTPP**	C_{2h}	D_4	D_{2d}
RMSD BD, Å[a]	3.E-04	4.E-04	0	RMSD BD, Å	1.E-04	2.E-04	0
N–Pt	2.03	2.03	2.03	N–Pt	2.03	2.03	2.03
$C_m\cdots C_m$	6.91	6.91	6.91	$C_m\cdots C_m$	6.89	6.89	6.89
N···N	4.06	4.06	4.06	N···N	4.05	4.05	4.05
C_a-C_m–C_1-C_2	67.7	68.8	65.3	C_a-C_m–C_1-C_2	82.6	84.4	80.1
C_b-C_b-C_a-N	0.4	0.7	1.1	C_b-C_b-C_a-N	0.2	0.2	0.4
C_a-N-N_{opp}-C_a	180.0	179.6	175.1	C_a-N-N_{opp}-C_a	180.0	179.9	178.4
C_a-N-N-C_a	0.7	0.3	4.1	C_a-N-N-C_a	0.2	0.1	1.3
C_b-C_a-N-Pt	178.1	179.8	174.5	C_b-C_a-N-Pt	179.4	179.9	178.1
C_m-C_m-C_m-C_m	0.0	0.0	0.0	C_m-C_m-C_m-C_m	0.0	0.0	0.0
C_b-C_b-C_b-C_b	0.0	0.6	0.0	C_b-C_b-C_b-C_b	0.0	0.1	0.0
RMSD Pl.,° [b]	0.8	0.5	4.2	RMSD Pl.°	0.3	0.1	1.3
B97D				B3LYP			
**$PtTF_5PP$**	C_{2h}	D_4	D_{2d}	**$PtTF_5PP$**	D_{4h}		
RMSD BD, Å[a]	8.E-05	1.E-04	0	RMSD BD, Å	0		
N–Pt	2.03	2.03	2.03	N–Pt	2.03		
$C_m\cdots C_m$	6.89	6.89	6.89	$C_m\cdots C_m$	6.87		
N···N	4.06	4.06	4.06	N···N	4.05		
C_a-C_m–C_1-C_2	74.9	75.3	74.2	C_a-C_m–C_1-C_2	90.0		
C_b-C_b-C_a-N	0.3	0.3	0.5	C_b-C_b-C_a-N	0.0		
C_a-N-N_{opp}-C_a	180.0	180.0	177.6	C_a-N-N_{opp}-C_a	180.0		
C_a-N-N-C_a	0.5	0.0	2.0	C_a-N-N-C_a	0.0		
C_b-C_a-N-Pt	178.1	179.8	174.5	C_b-C_a-N-Pt	0.0		
C_m-C_m-C_m-C_m	0.0	0.0	0.0	C_m-C_m-C_m-C_m	0.0		
C_b-C_b-C_b-C_b	0.0	0.5	0.0	C_b-C_b-C_b-C_b	0.0		
RMSD Pl.,° [b]	0.7	0.2	2.8	RMSD Pl.°	0.0		

[a] RMSD BD - root mean square deviation of all bond lengths of $PtTY_5PP$ Y: H, F, calculated relative to the structural parameters of conformer corresponding to the minimum on the PES (D_{2d});

[b] RMSD Pl. - root mean square deviation characterizing the difference of the macrocycle torsional angles (27 angles) from values 180/0°.

$$RMSD\ BD = \sqrt{\frac{\sum [(p_{i(D2d)} - p_{i(j\ conf)})]^2}{n}},\ RMSD\ Pl. = \sqrt{\frac{\sum [(\frac{180}{0} - i_{t.angle})]^2}{k}},\ n = 86, k = 27$$

Table S4. Geometrical parameters of PtTPP and PtTF$_5$PP by GED for two variants of the starting model

	GED r_{h1}				B3LYP r_e		B97D r_e	
	PtTPP B3LYP (D_{2d})	PtTPP B97D (D_{2d})	PtTF$_5$PP B3LYP (D_{4h})	PtTF$_5$PP B97D (D_{2d})	PtTPP (D_{2d})	PtTF$_5$PP (D_{4h})	PtTPP (D_{2d})	PtTF$_5$PP (D_{2d})
r(Pt–N)	2.024(4)	2.025(4)	2.021(4)	2.032(5)	2.027	2.026	2.032	2.030
r(C$_a$–N)	1.380(3)	1.383(3)	1.376(3)	1.378(3)	1.375	1.373	1.383	1.380
r(C$_a$–C$_b$)	1.446(3)	1.440(3)	1.446(3)	1.437(3)	1.439	1.439	1.440	1.440
r(C$_b$–C$_b$)	1.362(3)	1.359(3)	1.357(3)	1.354(3)	1.352	1.351	1.360	1.359
r(C$_m$–C$_a$)	1.398(3)	1.398(3)	1.394(3)	1.393(3)	1.393	1.391	1.399	1.395
r(C$_m$–C$_1$)	1.502(3)	1.491(3)	1.499(3)	1.489(3)	1.497	1.495	1.492	1.492
r(C$_1$–C$_2$)	1.401(3)	1.403(3)	1.395(3)	1.397(3)	1.396	1.392	1.403	1.399
r(C$_2$–C$_3$)	1.394(3)	1.396(3)	1.391(3)	1.395(3)	1.390	1.388	1.397	1.397
r(H–C$_b$)	1.085(5)	1.090(5)	1.076(3)	1.081(3)	1.075	1.076	1.080	1.081
r(C–C)$_{ave}$	1.396(3)	1.399(3)	1.392(3)	1.396(3)	1.392	1.389	1.399	1.398
r(C–F)$_{ave}$	–	–	1.336(3)	1.337(3)	–	1.334	–	1.340
a(Me-N-C$_a$)	127.2(2)	126.7(2)	127.4(2)	126.6(3)	126.6	126.6	126.7	126.8
a(C$_a$-N-C$_a$)	105.6(3)	106.4(3)	105.2(4)	106.7(6)	106.8	106.7	106.6	106.4
a(N-C$_a$-C$_b$)	110.4(3)	109.5(3)	110.8(1)	109.3(5)	109.3	109.4	109.4	109.6
a(C$_a$-C$_m$-C$_a$)	125.7(5)	124.5(1)	126.9(2)	124.7(1)	124.3	124.9	124.5	125.2
a(C$_1$-C$_2$-C$_3$)	120.6(4)	120.5(3)	122.1(1)	121.6(2)	120.7	122	120.6	121.9
Θ(C$_2$-C$_1$-C$_m$-C$_{a1}$)	89(167)	71(4)	90	79($_{-5}^{+27}$)[a]	80.1	90	65.3	74.2
R_f	4.6%	3.9%	5.8%	4.4%				

[a] The uncertainty in the torsion angle value for PtTF$_5$PP is determined using Hamilton's statistical criterion as described in Figure 4 (main text).

Table S5. Experimental and theoretical vibrational amplitudes l_{exp}, l_{theor} and corrections $r_{h1}-r_a$ for PtTPP (B97D start model)

Group of amplitudes for distances in a range, Å	Labels see on Figure S5		l_{exp}	l_{theor}	$r_{h1}-r_a$
0.00–1.20	69 70	C–H_{ave}	0.082(9)	0.076	0.0010
1.20–1.70	4 9	C–N	0.052(2)	0.050	0.0046
	16 20	C_m–C_{ph}	0.056(2)	0.053	0.0001
	8 16	C_m–C_a	0.050(2)	0.048	–0.0054
	69 70	C_b–C_b	0.047(1)	0.045	–0.0059
	21 25	C_{ph}–C_{ph}	0.051(2)	0.049	0.0003
1.70–2.10	1 3	Pt–N	0.066(7)	0.060	0.0090
2.10–2.50	7 8	$C_a\cdots C_a$	0.058(4)	0.056	0.0048
	8 20	$C_{Ph}\cdots C_a$	0.078(5)	0.075	–0.0012
2.50–2.75	15 27	$C_m\cdots C_{ph}$	0.094(11)	0.085	0.0077
	17 71	$C_m\cdots C_b$	0.079(9)	0.071	–0.0044
2.75–3.35	28 32	$C_{ph}\cdots C_{ph}$	0.079(5)	0.075	0.0077
3.35–3.70	9 24	$C_a\cdots C_{ph}$	0.162(12)	0.163	0.0084
3.70–4.00	26 73	$C_b\cdots C_{Ph}$	0.282(19)	0.265	0.0010
	4 20	$N\cdots C_{ph}$	0.079(6)	0.082	0.0161
4.00–4.68	5 3	N···N	0.091(4)	0.080	0.0130
	3 10	$N\cdots C_a$	0.104(5)	0.093	0.0293
4.68–5.53	15 18	$C_m\cdots C_m$	0.094(6)	0.078	0.0192
	11 41	$C_a\cdots C_{ph}$	0.18(11)	0.150	0.0220
5.53–6.20	35 68	$C_b\cdots C_{ph}$	0.32(21)	0.312	0.0086
	10 13	$C_a\cdots C_a$	0.099(7)	0.096	0.0416
6.20–6.60	14 17	$C_a\cdots C_m$	0.120(10)	0.109	0.0593
	6 70	$N\cdots C_b$	0.105(9)	0.095	0.0446
6.60–7.50	6 42	$N\cdots C_{ph}$	0.163(11)	0.149	0.0519
	16 18	$C_m\cdots C_m$	0.140(9)	0.116	0.0669
	1 38	$Pt\cdots C_{ph}$	0.199(13)	0.166	0.0707
7.50–7.80	9 22	$C_a\cdots C_{ph}$	0.135(16)	0.122	0.0827
	17 67	$C_m\cdots C_b$	0.132(16)	0.110	0.0636
7.80–9.70	27 71	$C_b\cdots C_{ph}$	0.310(40)	0.290	0.1187
9.70–11.80	42 49	C…H	0.959(185)	0.606	0.0526
11.80–20.00	23 33	$C_{ph}\cdots C_{ph}$	0.597(449)	0.467	0.2222
	61 59	H···H	0.377(283)	0.292	0.4109

Table S6. Experimental and theoretical vibrational amplitudes l_{exp}, l_{theor} and corrections r_{h1}–r_a for $PtTF_5PP$ (B97D starting model)

Group of amplitudes for distances in a range, Å	Labels see on Figure S5		l_{exp}	l_{theor}	r_{h1}–r_a
0.00–1.20	69 70	C–H_{ave}	0.084(64)	0.076	0.0014
1.20–1.70	4 9	C–N	0.052(2)	0.050	0.0039
	16 20	C_m–C_{ph}	0.056(2)	0.053	0.0002
	8 16	C_m–C_a	0.050(2)	0.048	–0.0043
	69 70	C_b–C_b	0.047(2)	0.045	–0.0048
	21 25	C_{ph}–C_{ph}	0.051(2)	0.049	–0.0002
1.70–2.10	1 3	Pt–N	0.067(14)	0.060	0.0086
2.10–2.65	7 8	C_a···C_a	0.059(3)	0.056	0.0045
	8 20	C_{Ph}···C_a	0.078(4)	0.075	–0.0012
2.65–3.06	28 32	C_{ph}···C_{ph}	0.079(8)	0.073	0.0084
	52 60	F···F	0.140(14)	0.128	0.0102
3.06–3.30	44 76	H···F	0.543(134)	0.549	0.0833
3.30–3.95	26 73	C_b···C_{Ph}	0.319(20)	0.296	0.0206
3.95–4.53	45 70	C_b···F	0.635(50)	0.472	0.0974
	5 3	N···N	0.090(8)	0.075	0.0174
	3 10	N···C_a	0.105(9)	0.088	0.0242
4.53–5.22	11 13	C_a···C_a	0.095(8)	0.080	0.0195
5.22–6.05	32 70	C_b···F	0.323(19)	0.341	0.0327
	10 13	C_a···C_a	0.085(5)	0.089	0.0348
6.05–6.60	6 70	N···C_b	0.097(12)	0.087	0.0368
	17 48	C_m···F	0.530(66)	0.475	0.0734
6.60–7.60	16 18	C_m···C_m	0.119(13)	0.108	0.0555
	70 52	C_b···F	0.555(59)	0.479	0.0620
7.60–7.90	9 22	C_a···C_{ph}	0.140(28)	0.113	0.0712
7.90–9.90	1 51	Pt···F	0.295(45)	0.244	0.0875
	27 71	C_b···C_{ph}	0.295(45)	0.265	0.0994
9.90–11.40	47 49	F···F	1.030(161)	0.868	0.0934
11.40–20.00	59 61	F···F	0.299(117)	0.256	0.3851

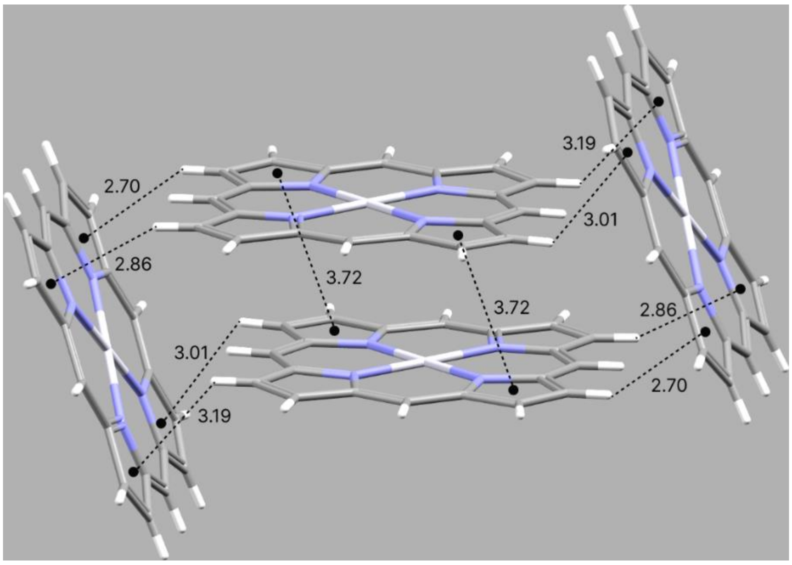

Figure S1. Black lines – aryl-stacking and CH-aryl interactions distance between the PtP molecules in the crystal. Refcode: USIVAV (distances in Å). Crystal density: 2.284 g cm^{-3}. Crystal system: monoclinic.

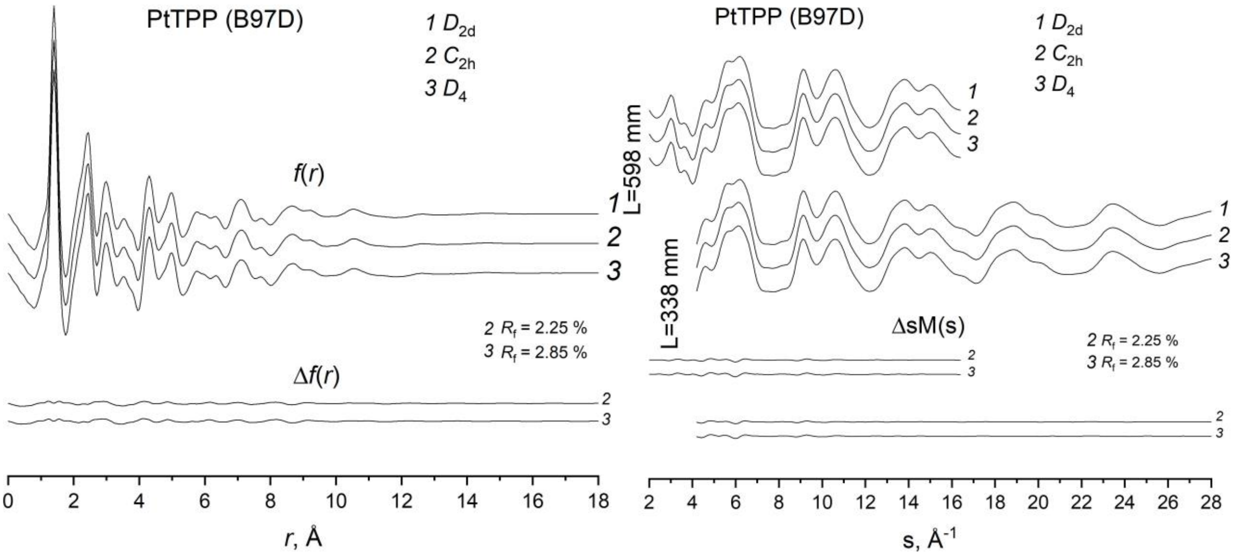

Figure S2. Comparison of theoretical *f*(*r*) and *sM*(*s*) of PtTPP conformer models according to B97D calculations

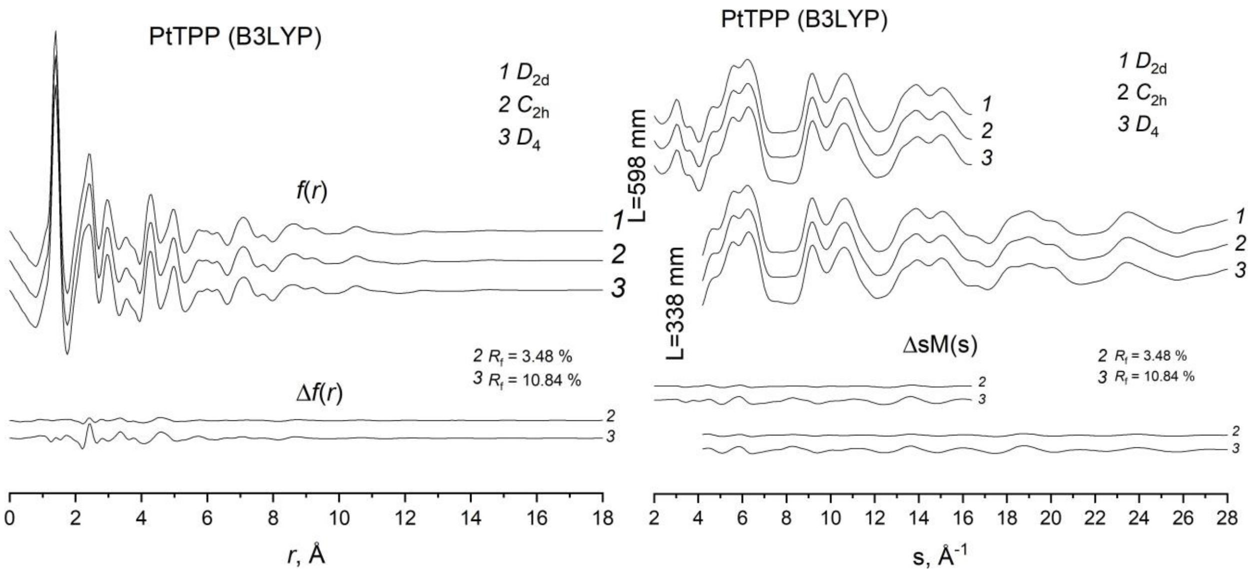

Figure S3. Comparison of the theoretical *f*(*r*) and *sM*(*s*) of the PtTPP conformer models according to B3LYP calculations

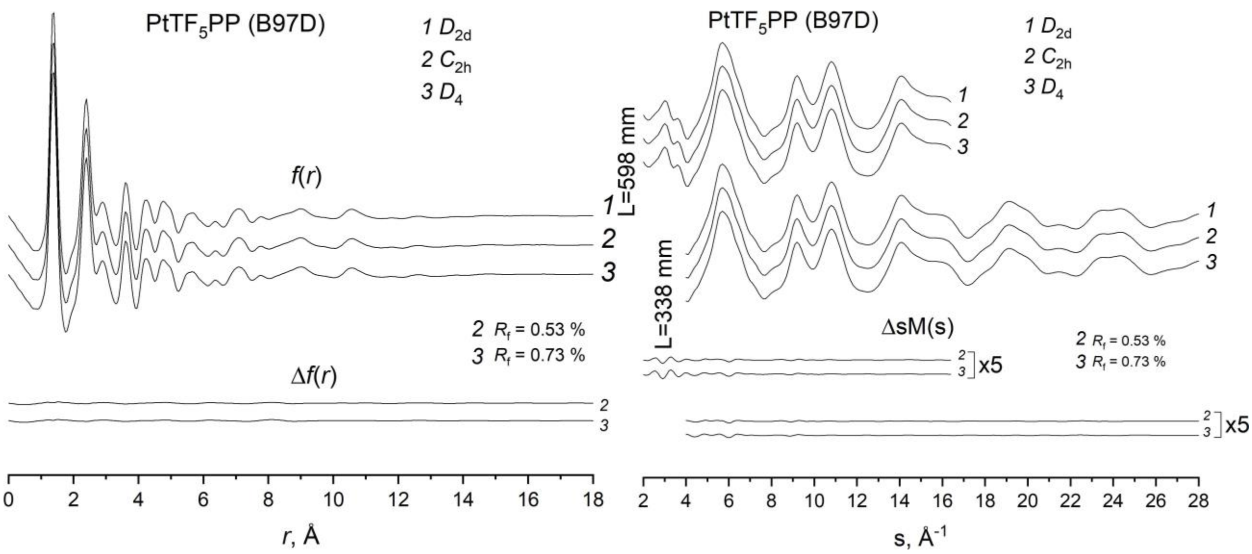

Figure S4. Comparison of the theoretical *f*(*r*) and *sM*(*s*) of the $PtTF_5PP$ conformer models according to B97D calculations

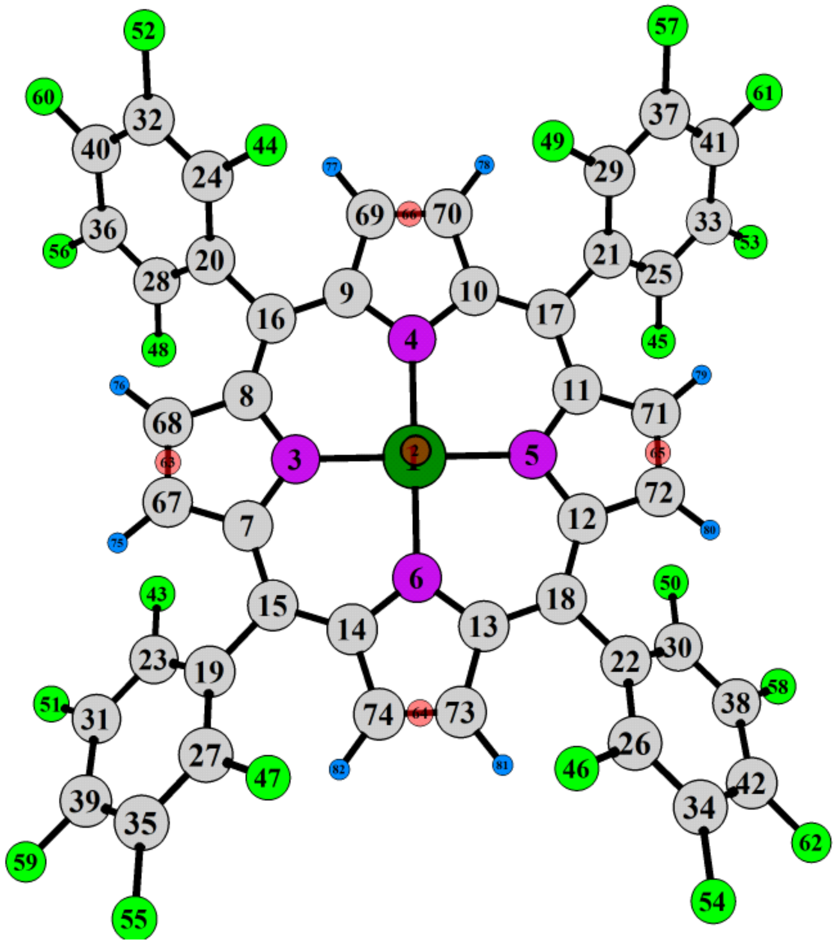

Figure S5. Atom labels of PtTPP and PtTF$_5$PP in Tables S5–S6.

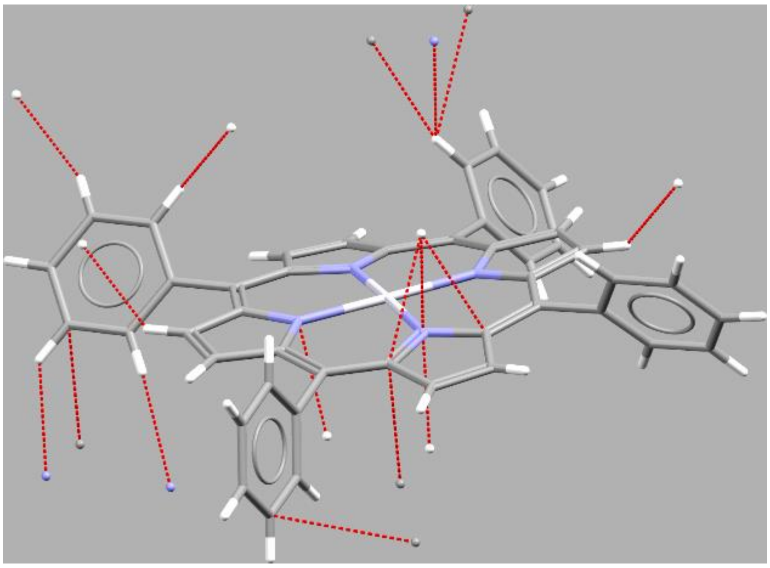

Figure S6. Single molecule in the PtTPP crystal. Refcode: CEZKEX01.

Red lines are shorter intermolecular contacts in the crystal smaller than the sum of the vdW radii. Crystal density: 1.704 g cm^{-3}. Crystal system: triclinic.

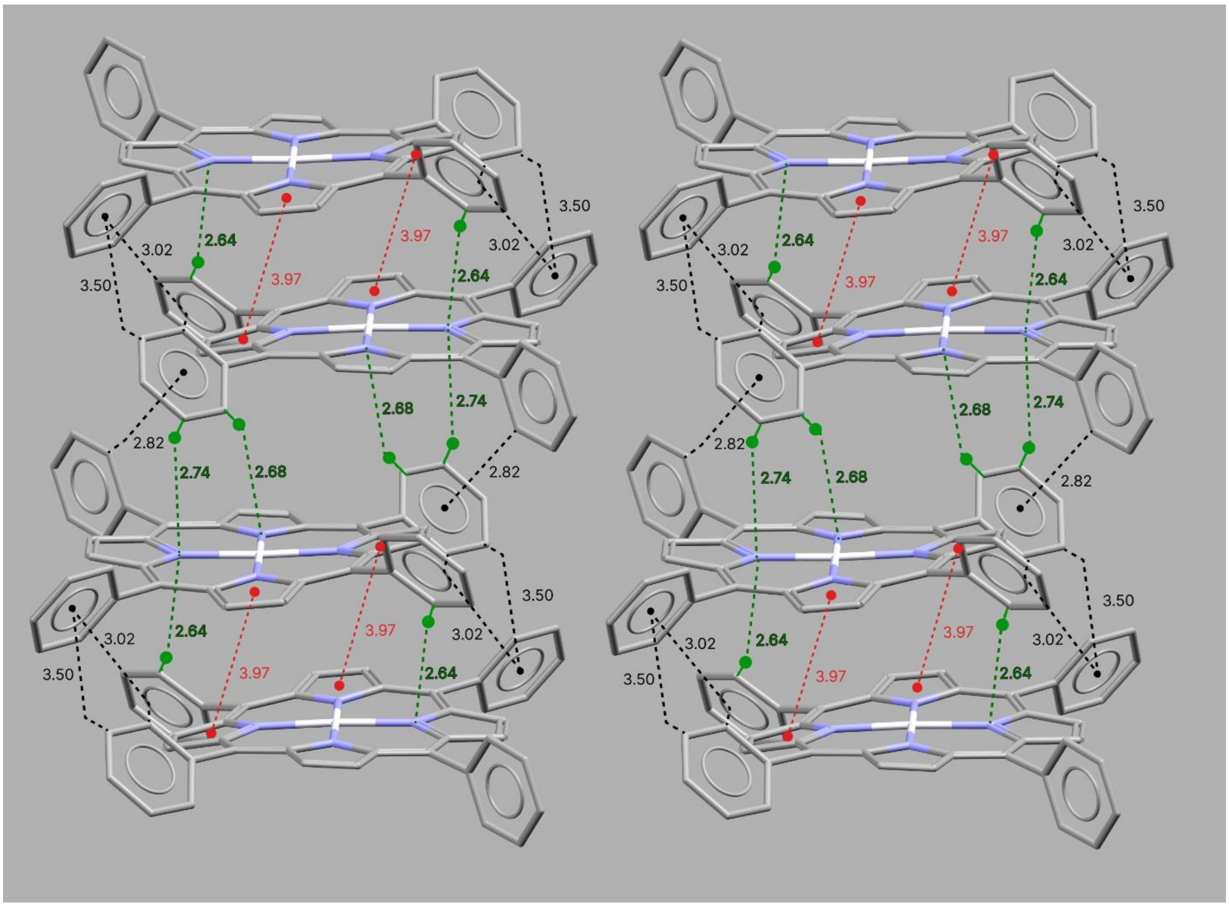

Figure S7. A fragment of the PtTPP crystal. Refcode: CEZKEX01 (distances in Å).

Black lines are T-shaped stacking interaction distance between the C_6H_5 rings; red lines are aryl-stacking interaction distances between the pyrrole rings; green lines are C-H···N interactions (the green circles indicate hydrogen atoms only, which enter into this interaction type). Crystal density: 1.704 g cm^{-3}. Crystal system: triclinic.

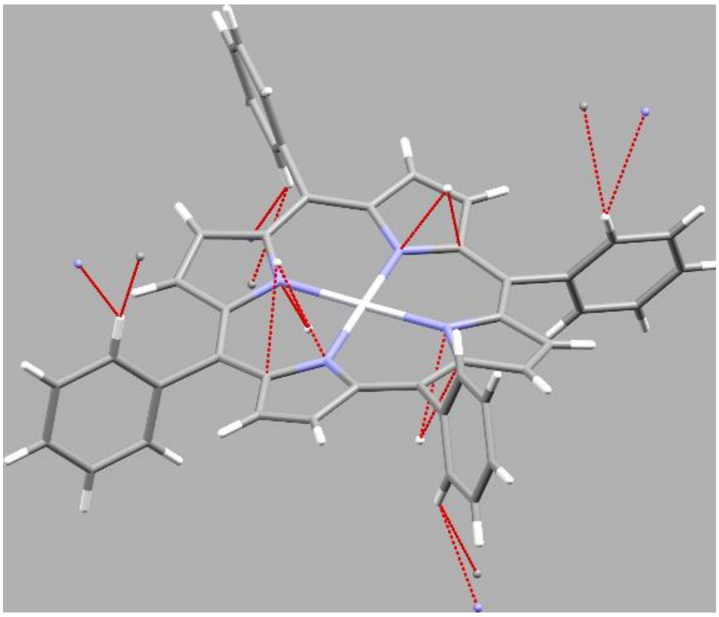

Figure S8. Single molecule in the PtTPP crystal. Refcode: CEZKEX02.

Red lines are shorter intermolecular contacts in crystal smaller than the sum of vdW radii. Crystal density: 1.734 g cm^{-3}. Crystal system: tetragonal.

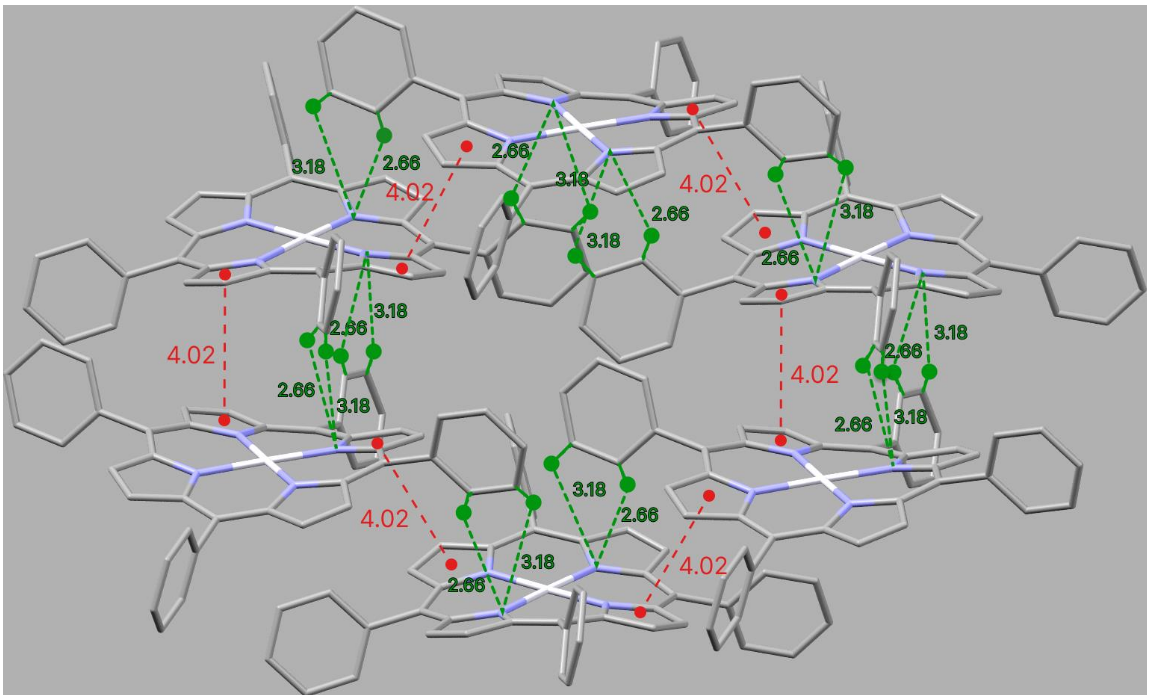

Figure S9. A fragment of the PtTPP crystal. Refcode: CEZKEX02 (distances in Å).

Red lines are aryl-stacking interaction distances between the pyrrole rings; green lines are C-H···N interactions (the green circles indicate hydrogen atoms, which are involved in this interaction type). Crystal density: 1.734 g cm^{-3}. Crystal system: tetragonal.

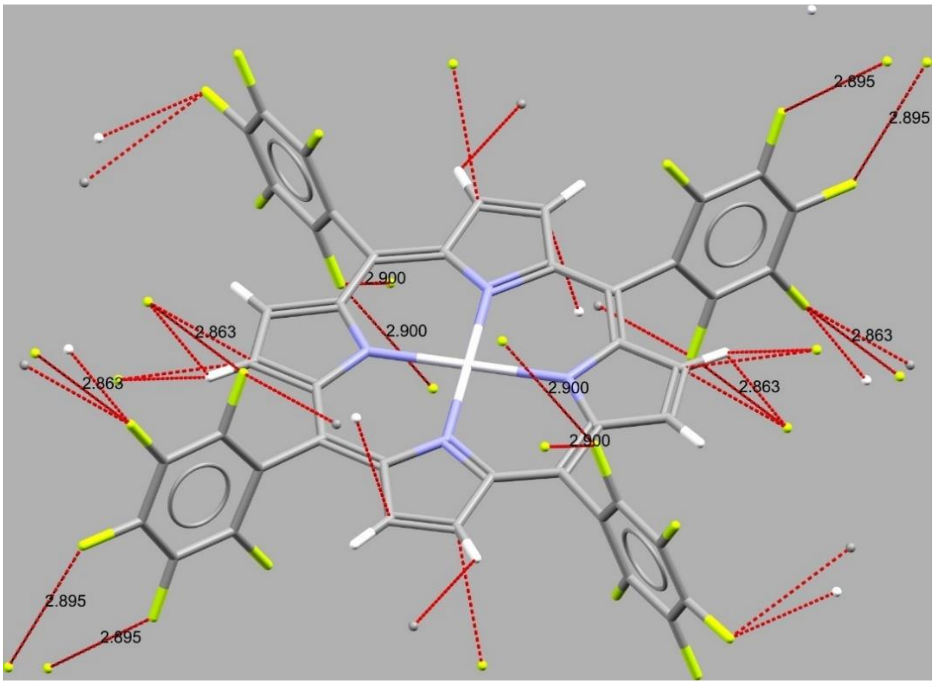

Figure S10. Single molecule in the PtTF$_5$PP crystal with measured F···F distances in Å. Refcode: CIQXUW (distances in Å).

Red lines are shorter intermolecular contacts in crystal smaller than the sum of vdW radii. Crystal density: 2.074 g cm^{-3}. Crystal system: monoclinic.

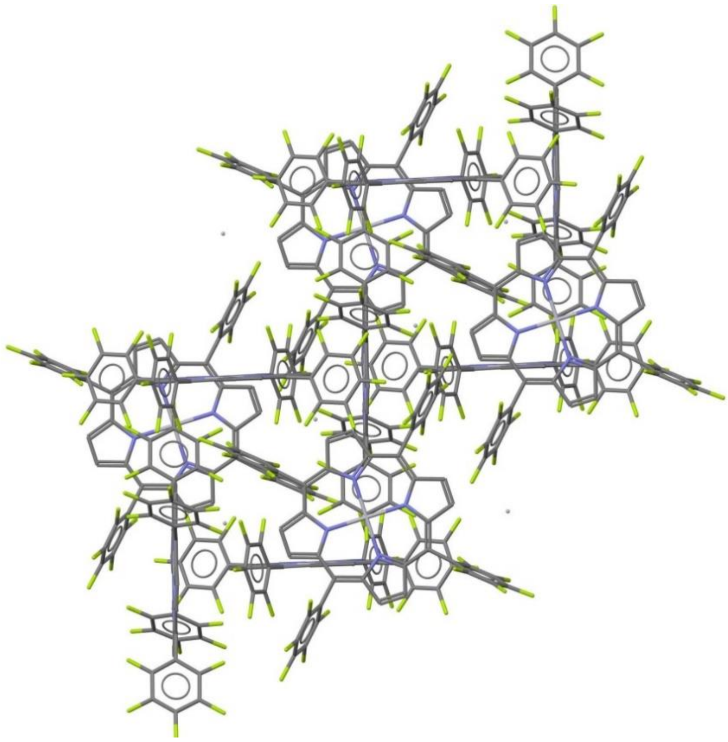

Figure S11. Unit cell of the $PtTF_5PP$ crystal. Refcode: CIQXUW. Crystal density: 2.074 g cm^{-3}. Crystal system: monoclinic.

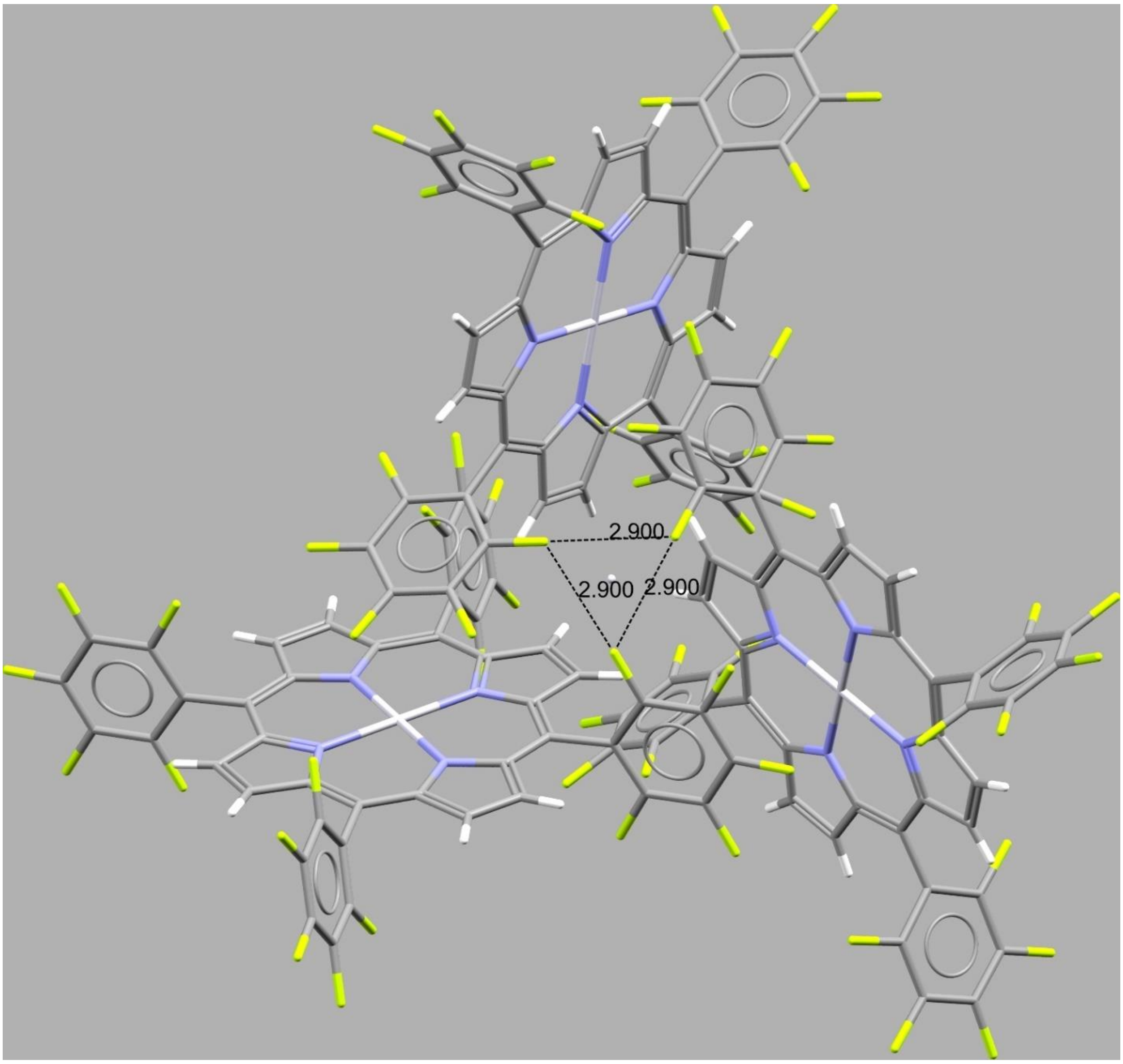

Figure S12. A fragment of the $PtTF_5PP$ crystal. Symmetric interactions between the fluorine atoms of neighboring molecules. Refcode: CIQXUW (distances in Å). Crystal density: 2.074 g cm^{-3}. Crystal system: monoclinic.

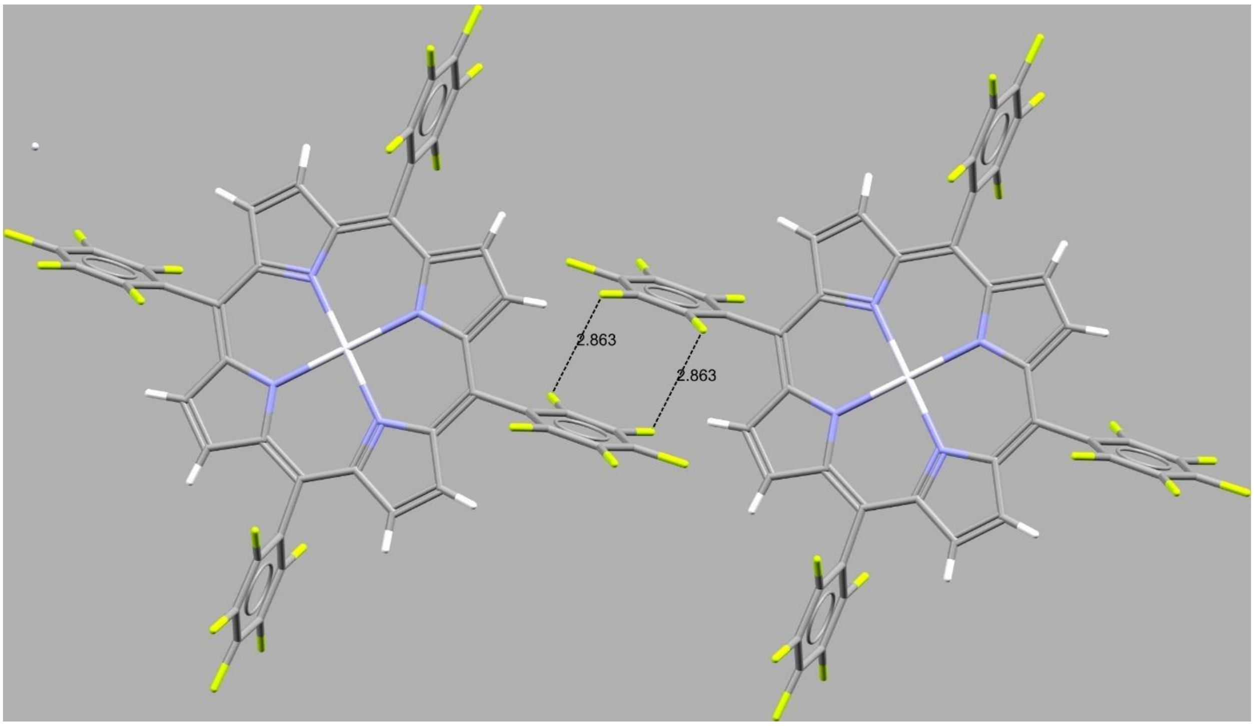

Figure S13. A fragment of the $PtTF_5PP$ crystal. Interaction between fluorine atoms of parallel oriented meso-substituents. Refcode: CIQXUW (distances in Å). Crystal density: 2.074 g cm^{-3}. Crystal system: monoclinic.
